# Serum Amyloid P Component (SAP)-Like Protein From Botryllid Ascidians Provides a Clue to Amyloid Function

**DOI:** 10.1155/1992/39710

**Published:** 1992

**Authors:** V. L. Scofield, L. Puntambekar, S. F. Schluter, D. R. Coombe

**Affiliations:** 1 Department of Biology UCLA School of Medicine Los Angeles California 90024 USA; 2 Department of Microbiology University of Arizona College of Medicine Tucson Arizona 85724 USA; 3 Sir William Dunn School of Pathology Oxford University South Parks Rd. Oxford OX1 3RE UK

**Keywords:** SAP, amyloid, pentraxin, protochordate, HA-1 lectin

## Abstract

The HA-1 lectin isolated from Botrylloides leachii has an amino acid composition similar to that of mammalian serum amyloid protein (SAP). SAP is a universal component of mammalian amyloid deposits. Like SAP, HA-1 has a disc ultrastructure, and antibody to HA-1 binds both (a) to amyloidlike fibers deposited between rejected *Botrylloides* colonies and (b) to cerebral amyloid deposits in Alzheimer's disease brains. Deposition of protochordate amyloid within rejection sites and surrounding fouling organisms implies that these fibers function as barriers to allogeneic and infectious challenge. Similarly, mammalian amyloid may also function to contain inflammatory lesions and to limit the spread of certain infections. Pathological amyloidotic conditions in humans, such as Alzheimer's disease, may result from unregulated expression of this primitive encapsulation response.


*Developmental Immunology*, 1992, Vol. 3, pp. 67–84
Reprints available directly from the publisher
Photocopying permitted by license only

© 1992 Harwood Academic Publishers GmbH
Printed in the United States of America

### Serum Amyloid P Component (SAP)-like Protein from Botryllid Ascidians Provides a Clue to Amyloid Function

V. L. SCOFIELD,†* L. PUNTAMBEKAR,† S. F. SCHLUTER,‡ and D. R. COOMBE§

†*Department of Biology, UCLA School of Medicine, Los Angeles, California 90024*
‡*Department of Microbiology, University of Arizona College of Medicine, Tucson, Arizona 85724*
§*Sir William Dunn School of Pathology, Oxford University, South Parks Rd., Oxford OX1 3RE, United Kingdom*

The HA-1 lectin isolated from *Botrylloides leachii* has an amino acid composition similar to that of mammalian serum amyloid protein (SAP). SAP is a universal component of mammalian amyloid deposits. Like SAP, HA-1 has a disc ultrastructure, and antibody to HA-1 binds both (a) to amyloidlike fibers deposited between rejected *Botrylloides* colonies and (b) to cerebral amyloid deposits in Alzheimer's disease brains. Deposition of protochordate amyloid within rejection sites and surrounding fouling organisms implies that these fibers function as barriers to allogeneic and infectious challenge. Similarly, mammalian amyloid may also function to contain inflammatory lesions and to limit the spread of certain infections. Pathological amyloidotic conditions in humans, such as Alzheimer's disease, may result from unregulated expression of this primitive encapsulation response.

KEYWORDS: SAP, amyloid, pentraxin, protochordate, HA-1 lectin.

### INTRODUCTION

For some years, animals belonging to the protochordate genera *Botryllus* and *Botrylloides* have been the subjects of intensive study by immunologists concerned with the evolutionary origins of the vertebrate immune response (Weissman et al., 1990). These colonial ascidians grow on hard substrata in communities where space is a limited resource and where colonies frequently come into contact. When this happens, juxtaposed colonies undergo vascular fusion to produce a genetic chimera or initiate rejection reactions with associated necrosis at the contact site in the tissues of each partner. Fusion or rejection is controlled by a single polymorphic histocompatibility gene locus (Oka and Watanabe, 1957; Scofield et al., 1982); fusion occurs when one allele at this locus is shared, whereas rejection follows contact between colonies sharing no alleles.

In an examination of the ultrastructure of necrotic areas in the contacted tissues between rejecting colonies of *Botryllus primigenus*, Tanaka and Watanabe (1973a, 1973b) described accumulations of stiff, hollow fibers with a characteristic diameter of around 200 angstroms. These ultrastructural features are reminiscent of polymerized serum amyloid P component (SAP), which is a universal component of mammalian amyloid deposits, and of a type of fiber observed in electron micrographs of some such deposits (Skinner et al., 1982; Inoue et al., 1986). Like the closely related C-reactive protein (CRP), SAP is a member of the highly conserved *pentraxin* family of serum and tissue proteins (Coe, 1983). Molecules belonging to this family have been identified in several representative vertebrate classes and in an invertebrate of the protostome lineage, the horseshoe crab *Limulus* (Robey and Liu, 1981). Until now, no pentraxin molecule has been identified from a deuterostome invertebrate.

The pentameric HA-1 lectin isolated from the hemolymph of *Botrylloides leachii* (Coombe et al., 1984a; Schluter and Ey, 1989) has subunit molecular weight and amino acid composition characteristics similar to those of prototypical vertebrate pentraxins (Schluter and Ey, 1989). Because of these similarities and the suggestive

*Corresponding author.

ultrastructure of fibers deposited between rejected *Botryllus* colonies (Tanaka and Watanabe, 1973a, 1973b), we set out to determine (a) whether HA-1 shows the disc ultrastructure of vertebrate pentraxins, (b) whether rejection areas and their contained fiber structures contain HA-1, and (c) whether antibodies to HA-1 identify cross-reacting determinants in mammalian amyloid deposits. Here we report (a) that HA-1 molecules show a typical pentraxin ultrastructure upon examination with the electron microscope after negative staining; (b) that antibodies to HA-1 show concentrated binding to tunicate rejection areas by light immunohistochemistry, and exhibit specific binding to rejection fibers by immunoelectron microscopy; and (c) that antibodies to HA-1 bind to mammalian amyloid in patterns identical to those of anti-SAP on the same material. The evidence presented suggests that allogeneic rejection in tunicates initiates a true amyloidotic process. Further, the concentration of amyloidlike fiber masses within rejection barriers, and the presence of HA-1 epitopes in these regions, suggests that the fiber network functions for separation of inflammatory foci from surrounding healthy tissue. These observations have interesting implications for the function of amyloid in mammals.

### RESULTS

### HA-1 Has Pentraxin Characteristics

**The amino acid composition of *Botrylloides* HA-1 molecules is similar to those of other vertebrate pentraxins.** HA-1 is a pentameric molecule whose identical disulfide-bonded subunits have a molecular weight similar to that of a prototypical pentraxin subunit (around 28,000; Coe, 1983; Coombe et al., 1984a; Schluter and Ey, 1989). In the present study, the blocked N-terminus of HA-1 necessitated cleavage and separation of the cleaved fragments prior to attempts at N-terminal amino acid sequencing. Even after these steps were taken, however, single amino acids could not be assigned confidently to each position (not shown). It is noteworthy that this result is reminiscent of similar findings with the *Limulus* pentraxin (Nguyen et al., 1986a, 1986b), which exhibits microheterogeneity between subunits giving rise to multiple assignments upon sequencing. The *Limulus* sequences were resolved eventually by sequencing of the three *Limulus* pentraxin genes (Nguyen et al., 1986b). Cloning and sequencing of the *Botrylloides* nucleotide sequences encoding HA-1 subunits will be required for definitive assignment of the HA-1 lectin to the pentraxin family of proteins (Coe, 1983). However, comparison of the amino acid composition data in Table 1 to vertebrate pentraxins reveals marked similarities to SAP/CRP molecules from other species (Skinner et al., 1980; Liu et al., 1982; Pepys et al., 1982). In addition, the ultrastructure of the HA-1 molecule provides strong evidence that it is related to mammalian CRP and SAP (see what follows).

**The disc structure of HA-1 is pentraxinlike.** Despite the wide phylogenetic separation of mammals, fish, and ancient arthropods, a unique property of vertebrate pentraxins that has been retained is the disclike form of their molecules in the electron microscope after negative staining (Fernandez-Moran et al., 1968; Pepys et al., 1978; Coe, 1983). Figure 1 shows that the HA-1 molecule has this characteristic. Both the wide-field and enlarged images of HA-1 in this figure show structures with subunits arranged as discs with a

TABLE 1
Amino Acid Composition of HA-1 Compared to Those of Other Pentraxins[a]

| Amino acid[b] | Residues/mole | | | |
|---|---|---|---|---|
| | HA-1 | pSAP[c] | hSAP[d] | lCRP[e] |
| Asp | 24 | 23 | 14 | 23 |
| Glu | 33 | 16 | 22 | 28 |
| Ser | 27 | 25 | 14 | 15 |
| Gly | 72 | 22 | 16 | 20 |
| His | 5 | 5 | 5 | 11 |
| Arg | 12 | 8 | 9 | 5 |
| Thr | 13 | 11 | 7 | 14 |
| Ala | 17 | 10 | 8 | 9 |
| Pro | 6 | 7 | 14 | 6 |
| Tyr | 3 | 6 | 12 | 5 |
| Val | 6 | 16 | 15 | 14 |
| Met | 4 | 3 | 1 | 2 |
| Ile | 10 | 10 | 11 | 12 |
| Leu | 16 | 12 | 16 | 21 |
| Phe | 6 | 9 | 12 | 8 |
| Lys | 9 | 8 | 11 | 12 |

[a]An aliquot of HA-1 was evaporated to dryness, hydrolyzed in 6 M HCl, and derivatized for amino acid analysis by reverse-phase HPLC (Bidlingmeyer et al., 1984).
[b]HA-1 has 259 amino acid residues.
[c]pCAP: plaice SAP composition (Pepys et al., 1982).
[d]hSAP: human SAP composition (Skinner et al., 1980).
[e]lCRP: *Limulus* CRP subunit B (Liu et al., 1982).

hole in the center. Like mammalian and fish SAP, some of the HA-1 molecules are loosely arrayed in chains or stacks. Two characteristics of the molecules in Fig. 1, however, are different from those of vertebrate pentraxins. First, although most of the molecules appear to have five subunits, some, like the *Limulus* molecule (Fernandez-Moran et al., 1968), have six. Second, although many of the HA-1 discs in Fig. 1 appear to display the 100-angstrom diameter typical of vertebrate pentraxins (Pepys et al., 1978), the other half appears to have a diameter of 220 angstroms (Fig. 1 and Fig. 1 inset), which is the diameter of the amyloidlike fibers deposited in rejection regions between *Botryllus* and *Botrylloides* oozooids (see what follows). The HA-1 preparation used in these studies is electrophoretically homogeneous under reducing conditions (Schluter and Ey, 1989), and thus the observed differences in subunit and molecular diameter are not likely to be due to the presence of a second subunit of a different molecular weight. Mammalian SAP occurs in the serum as paired pentameric discs that, like the HA-1 molecules in Fig. 1, tend to form stacks (Pepys et al., 1978; Coe, 1983). The twofold size difference between HA-1 images in Fig. 1 may reflect stacking by some of the molecules, or alternatively may be composed of dimeric or multimeric subunits.

### Protochordate Fiber Deposits Have Amyloid Histochemistry and Ultrastructure

**Birefringent deposits form between rejected oozooids.** Fused and rejected pairs of live *Botrylloides* oozooids are shown in Figs. 2(a) and 2(b). After the initial fusion of tunics between contacted oozooids, the interactants either complete the establishment of shared blood vessels leading to fusion (Katow and Watanabe, 1980), Fig. 2(a), or initiate rejection reactions that lead to reseparation (Tanaka and Watanabe, 1973a, 1973b; Katow and Watanabe, 1980; Scofield and Nagashima, 1982), Fig. 2(b). As in *Botryllus*, both the fusion and rejection reactions between juxtaposed *Botrylloides* oozooids are completed within 12 hr of first contact. Reseparation of rejected oozooids is accompanied by appearance of an opalescent mass of birefringent fibers in the tunic throughout the contact zone (Tanaka and Watanabe, 1973a, 1973b), Fig. 2(b).

**The fibers are stained by aniline dyes.** To determine whether ascidian amyloidlike fibers exhibit the histochemical characteristics of vertebrate amyloid, fiber deposits in sections from methacrylate-embedded oozooid pairs were subjected to standard histological tests for amyloid. Certain dyes, particularly fuchsin and Congo

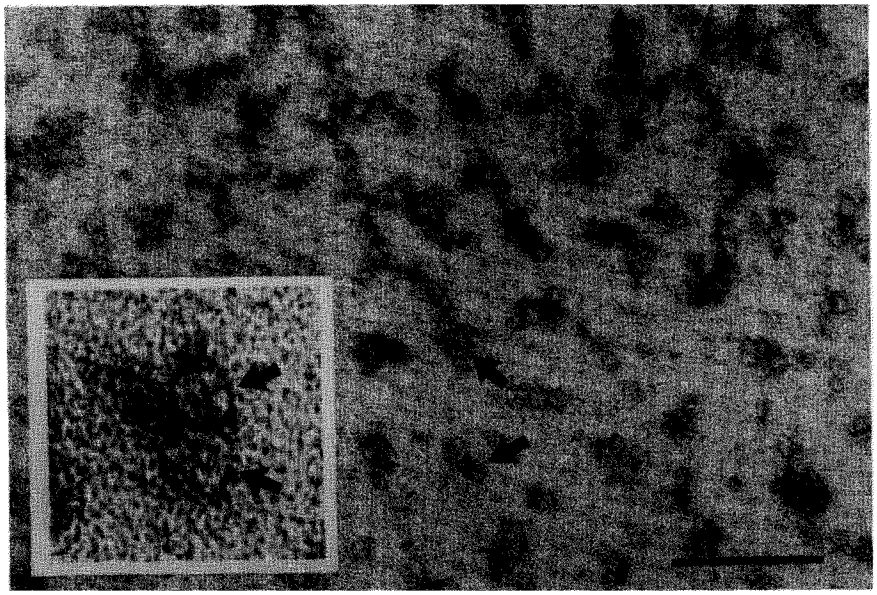

FIGURE 1. Electron micrograph of negatively stained *Botrylloides* leachii HA-1 molecules (Coombe et al., 1984a; Schluter and Ey, 1989). Most molecules are oriented face up (arrows), and many are aligned in rows (top arrow). Individual molecules appear to be pentameric or hexameric discs with a central hole (inset), and show apparent diameters of 100 and 220 nm (arrows, and inset). The bar spans 1500 nm.

Red, show remarkable affinity for amyloid (Skinner et al., 1982). Congo Red binds to amyloid fibers via hydrophobic interactions with proteins folded as beta-pleated sheets, and fuchsin dyes similarly show specific binding to amyloid fibers, but by an unknown mechanism (Puchtler et al., 1982). The regular alignment of dye molecules along the fibrils amplifies their intrinsic birefringence (Skinner et al., 1982). Figure 3 shows a section through a rejected *Botryllus* oozooid pair that has been stained with basic fuchsin and photographed under cross-polarized light. A wide birefringent wall of fibers cleanly bisects the area of joined tunic between the oozooids; see Fig. 3(a). A Congo Red-stained section is shown under higher magnification in Figs. 3(b) and 3(c) which are of the same field photographed under two different polarizing prism positions. When illuminated by cross-polarized light, the fiber masses composing the rejection "barrier" in these photographs, and in the inset showing a similar field in a replicate section, show the "apple-green" color and birefringence characteristics of Congo Red-stained amyloid deposits in mammals.

**The ultrastructure of fibers in rejection areas is similar to that of stacked SAP.** Reports

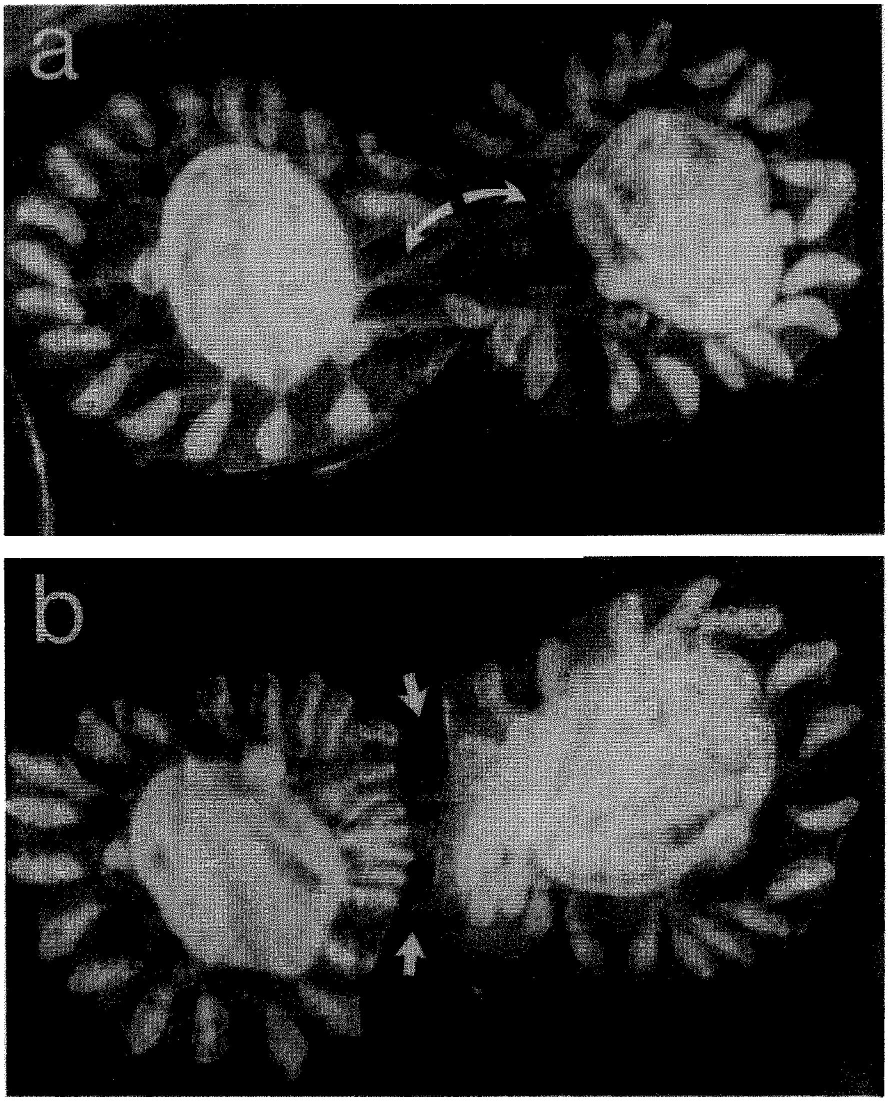

FIGURE 2. Fused and rejected *Botrylloides* oozooids pairs. (a) Fused oozooids. The arrows span one of three anastomosed blood vessels connecting the circulatory systems of the two zooids. (b) Rejected oozooids. Arrows point to the rejection area. A birefringent fiber deposit is visible in the facing tunic of the zooid on the right. Magnification ×50. (See Colour Plate I at the back of this publication).

detailing the ultrastructure of mammalian amyloid deposits frequently include descriptions of stiff, hollow rodlike fibers whose cross-sectional morphology is suggestive of stacked SAP molecules (Holck et al., 1979; Inoue et al., 1986; Miyakawa, 1988). A high-magnification transmission electron micrograph of fibers in rejection areas, like those in Figs. 3(a) to 3(c), is presented in Fig. 4. The 200-angstrom-wide fibers appearing in this electron micrograph are similar in ultrastructure and diameter and to those described by Tanaka and Watanabe (1973a, 1973b) in rejecting *Botryllus primigenus* colonies. Where bundles of fibers criss-cross in and out of the plane of section, those appearing in cross-section are hollow and appear to be pentameric or hexameric, and to contain a central hole. Structures of similar size and appearance have been described in the kidney mesonephros of lampreys (Ellis and Youson, 1989) and have also reported to be present in mammalian connective tissue (Inoue and Le Blond, 1986). Where they are described in mice, the fibers are immunoreactive with anti-SAP antibody (Inoue et al., 1986).

### Antibody to HA-1 Binds to Protochordate Amyloidlike Fibers

**HA-1 determinants are concentrated in rejection areas.** The ground substance of the tunicate tunic is composed of *tunicin*, a celluloselike polymer (Deck et al., 1966). Birefringence patterns in tunicate tissues stained with Congo Red or fuchsin, both of which are cellulose dyes (Puchtler et al., 1962), may not be specific markers for amyloidlike material in ascidian tissues. Instead, the presence of immunoreactive SAP is now considered to be a better diagnostic indicator for human amyloid (Pepys, 1988). If ascidian rejection fibers are structurally similar to mammalian amyloid, and if the HA-1 lectin is SAP-like in function as well as morphology, the HA-1 protein should be concentrated in the rejection area and present as a structural component of the

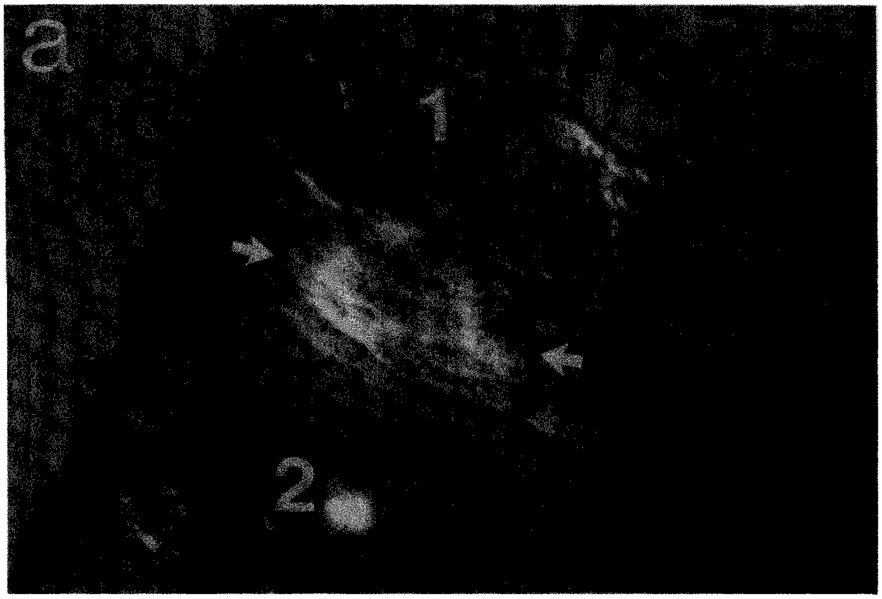

FIGURE 3. Fiber masses separating rejected *Botryllus* oozooids. (a) Birefringent fiber deposits form a barrier between rejected oozooids. This section was stained with fuchsin and viewed under cross-polarized light. A wide "fence" of birefringent fibers (denoted with arrows) separates zooids 1 and 2. ×50. (b) and (c) Fiber deposits exhibit Congophilic birefringence. Section similar to that in (a) stained with Congo Red and viewed under higher magnification with cross-polarized light. Fibers deposited between zooids 1 and 2 are shown under left- and right-side illumination by the polarization filters, allowing visualization of the phase change (compare fibers denoted by large arrow in (b) and (c) and the green color, arrow in (c) of the birefringent deposits). Tissues of zooid 1 are visible at the lower left in (b), and an ampulla of zooid 2 is visible in (c) ("amp"). Magnification: ×400. Inset: Another field between rejected oozooids in a similar section, showing lighter (small arrows) and darker (large arrows) green birefringence of deposits between the zooids. Magnification: ×400. (See Colour Plate II at the back of this publication).

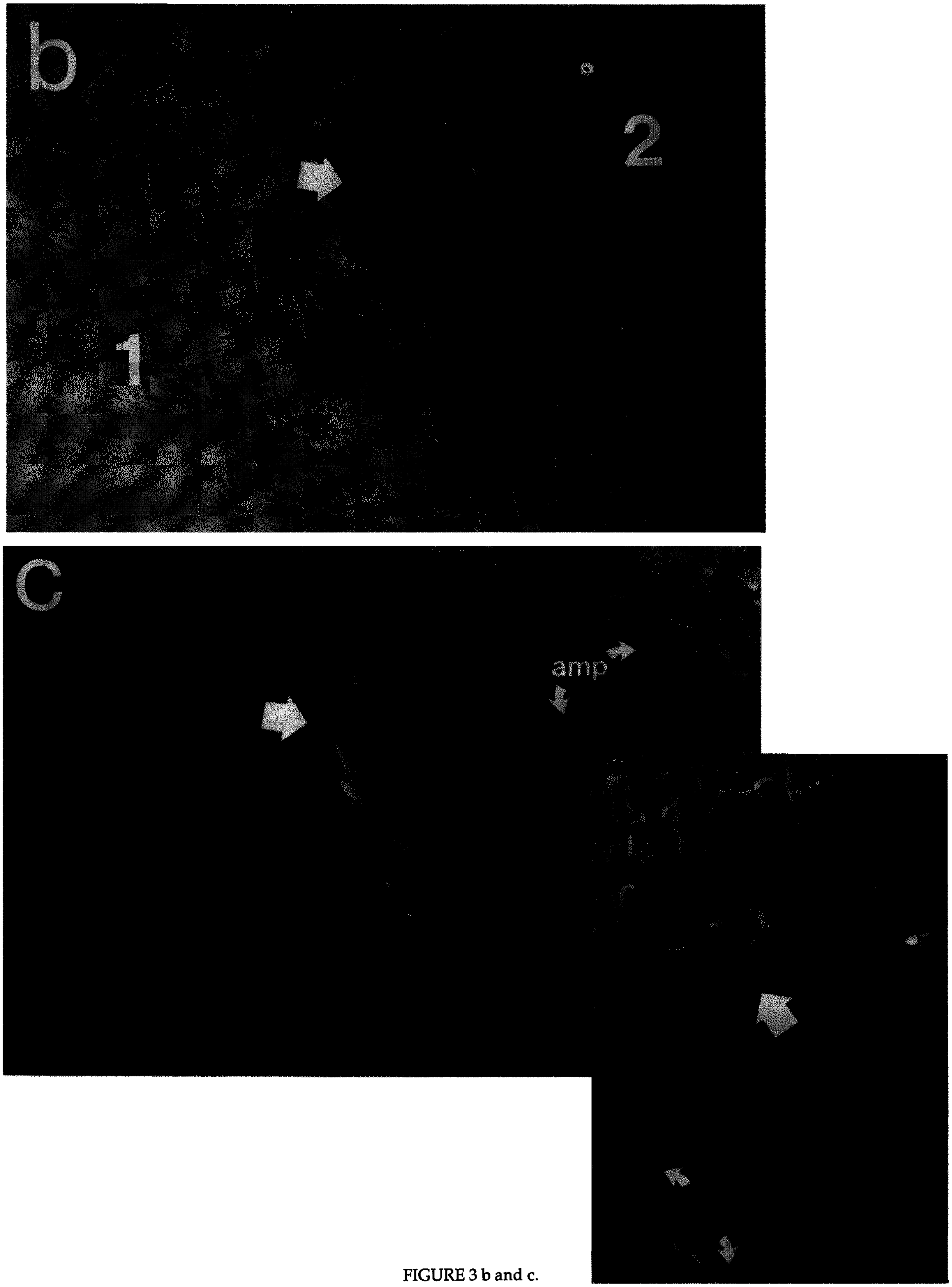

FIGURE 3 b and c.

double-tracked fibers in these locations. We therefore used the rabbit antibody to HA-1 (Coombe et al., 1984b) to test for the presence and location of HA-1 epitopes in oozooid rejection areas and rejection fibers, and to identify HA-1 epitopes in other body tissues.

Figure 5 shows replicate methacrylate sections taken through a rejected *Botrylloides* oozooid pair, incubated with normal rabbit serum, anti-SAP or anti-HA-1. Whereas neither the normal rabbit serum nor the antihuman SAP displayed significant binding to these sections (as evidenced by failure of goat antirabbit Ig and the Avidin-D and Protein A-labeled Fluoresbrite beads to identify sites of bound antibody on the sections in Figs. 5 (a) to 5 (d)), the anti-HA-1 bound to all the zooid tissues, with the heaviest labeling in the contact zone (Figs. 5(e) and 5(f)).

**Anti-HA-1 binds to fibers in rejection regions.** Figure 6 shows different areas from thin sections taken through birefringent areas of Epon-embedded rejected *Botrylloides* oozooids. Figure 6(a) shows both the tunic cuticle and an area underlying it where debris from degenerating test cells is surrounded by double-tracked fibers, as described by Tanaka and Watanabe (1973a, 1973b). Binding of anti-HA-1 to fibers within these areas is clearly evident in Fig. 6(a) and in the magnified area from this photograph shown in 6(b). In Fig. 6(c), heavy gold labeling marks the area underlying a cnidarian polyp embedded in the tunic. As in Fig. 5, neither normal rabbit serum nor anti-SAP showed significant binding to tunicate tissues in similar sections (Figures 7 and 8).

### HA-1 Tissue Distribution Is Similar to That of CRP and SAP in Mammals

**HA-1 determinants are present on blood cells, in basement membranes, and in endostylar tissues.** By immunoelectron microscopy, it was apparent that anti-HA-1 bound to *Botrylloides* cells and tissues in a pattern suggesting the blood cell and tissue distribution of SAP in mammals (Fig. 7). Previous studies have shown that anti-HA-1 binds to the macrophagelike granular amoebocytes from *Botrylloides* hemolymph (Coombe, 1983). In mice and humans, monomeric SAP and CRP are present on B-cell and NK-cell surfaces (Bray et al., 1988; James et al., 1983) and SAP occurs as a receptor-bound surface molecule on macrophages (Siripont et al., 1988). In addition, SAP is present in endothelial basement membranes (Dyck et al., 1980), and is a component of elastic fibers in the skin and connective tissues of lower chordates and mammals alike (Inoue and LeBlond, 1986; Inoue et al., 1986; Ellis and Youson, 1989). Figure 7 shows that HA-1, like mammalian SAP, is apparently present on (and in) some blood cells, and on and in both the

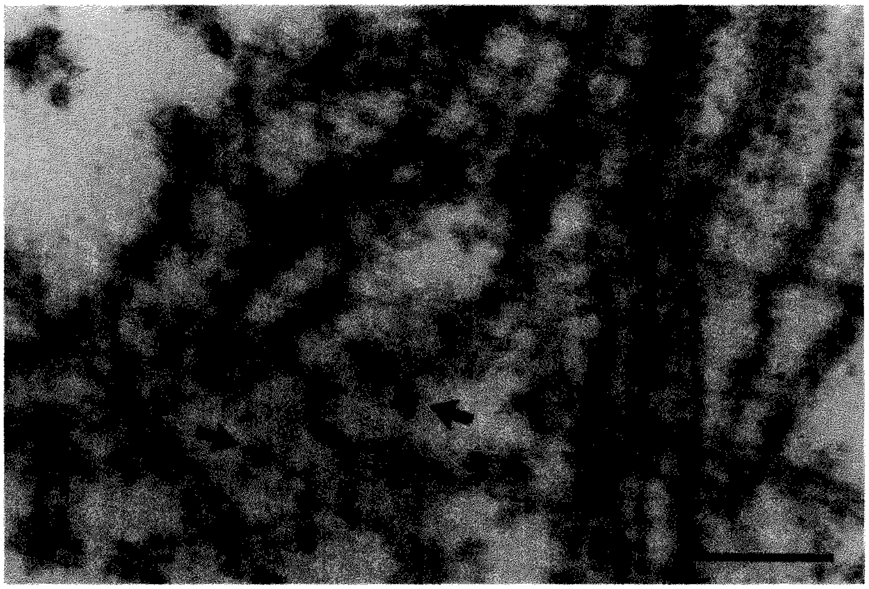

FIGURE 4. Transmission electron micrograph of deposits between rejecting *Botryllus* oozooids.The 150–200-angstrom-wide fibers are double tracked and show a regular periodicity of structure suggestive of stacked subunits. Fibers crossing the plane of section head on are pentameric or hexameric with a central hole, similar in shape and size to the larger HA-1 discs in Fig.1 and to stacked SAP (Pepys et al., 1978). The bar spans 1500 nm.

epithelial cells of blood vessel walls, with epitopes also extending into the surrounding microfibrillar network. In addition, it appears that HA-1, like mammalian CRP (Braun et al., 1986), may be present both in the blood and in seromucous fluids.

Tunicates are filter-feeding organisms, and have a ciliated epithelial structure called the *branchial basket* that both supplies particle-trapping mucus and rolls it into a tube for digestion in the gut (Goodbody, 1974). The rolling is done by specialized elongated *languet* cilia inside the thyroidlike endostyle (a structure that is also present in larval lampreys; Ellis and Youson, 1989). Figures 8(a) to 8(c) show that HA-1 determinants are concentrated heavily on surfaces of the languet cilia, where they may be components either of the ciliary membrane or of the mucus that bathes these structures in the living animal. That the latter possibility is more likely is suggested by the presence of concentrated HA-1 determinants in the membranes of endostylar cell

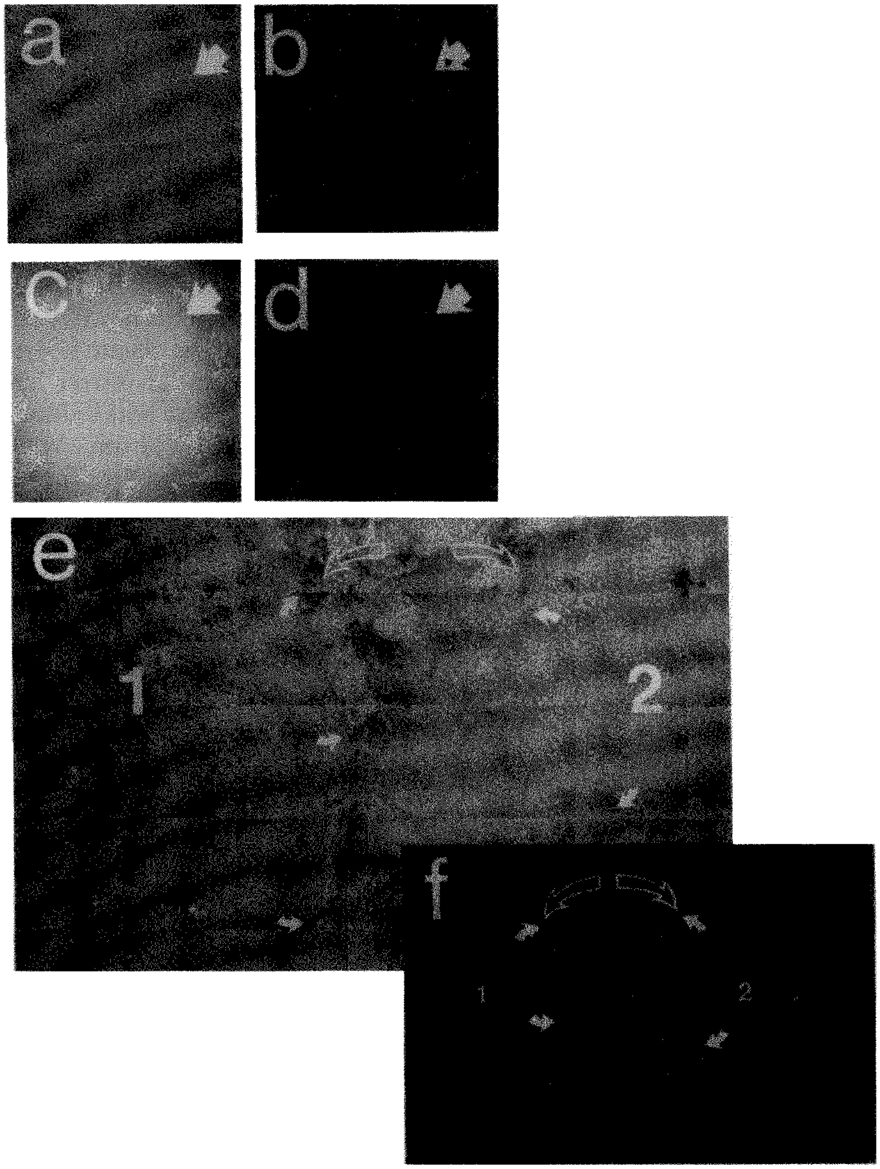

FIGURE 5. Binding by anti HA-1 to the contact zone between rejected oozooids. After incubation with normal rabbit serum, anti SAP or anti HA-1, sections were incubated first with biotinylated goat antirabbit immunoglobulin and then with a mixture of avidin-D coupled and Protein A-coupled Fluoresbrite beads. (a) and (b): Bright-field and dark-field photographs of a section incubated with normal rabbit serum. The section is negative for antibody binding. (c) and (d) section incubated with anti SAP. Some fluoresbrite beads are bound to the section, but no binding pattern is evident. Magnification: ×10. (e) and (f) Serial sections with methylene blue (E) or incubated with anti-HA-1 (F). In (e) zooids 1 and 2 are separated by a necrotic rejection region (spanned by hollow arrows). Small arrows in zooid 1 mark its rejection zone, and in zooid 2 circumscribe a much larger rejection area filling the tunic to the right of the original contact site. Magnification: ×63. In (f), these outlines are also evident in the areas of concentrated anti-HA-1 binding on a replicate section. The crisp profile of the zooid 1 rejection line and the broad rejection zone of zooid 2, from (e), are clearly outlined in Fluoresbrite beads in (f). Magnification: ×25. (See Côlour Plate III at the back of this publication).

secretory vacuoles, as well as on the surfaces of ciliated epithelial cells from other areas of the branchial basket; Figs. 8(d) and 8(e).

### Anti-HA-1 and Anti-SAP Bind to Alzheimer's Disease Amyloid in Identical Patterns

In mammals, SAP is a universal component of amyloid deposits, including the cerebral amyloid deposits of Alzheimer's disease (Coria et al., 1988; Pepys, 1988). To determine whether HA-1 and mammalian SAP might share antigenic determinants, we first tested anti-HA-1 and anti-SAP against purified HA-1, SAP, and CRP by peroxidase–anti-peroxidase enzyme-linked immunosorbent assays (ELISA) and by immunoblotting. For both antibodies, positive reactions were observed only with the homologous proteins (not shown). On the other hand, SAP present in formed amyloid deposits might display conserved epitopes not accessible on the native molecules (James et al., 1983; Maudsley and Pepys, 1987; Bray et al., 1988). To test mammalian amyloid for the presence of HA-1 cross-reactive determinants, tissue was obtained from the brains of patients who had died with Alzheimer's disease.

Unlike amyloidotic tissue from other locations, the cerebral amyloid of Alzheimer's disease brains has a finely specialized pattern of deposition into three sites: the walls of microvessels, focal plaque cores, and halos of degenerating neurites surrounding the cores (Vinters and Gilbert, 1983; Coria et al., 1988) (Fig. 9). Incubation of Alzheimer's brain tissue with anti-SAP followed by fluorescent detection reagents reveals

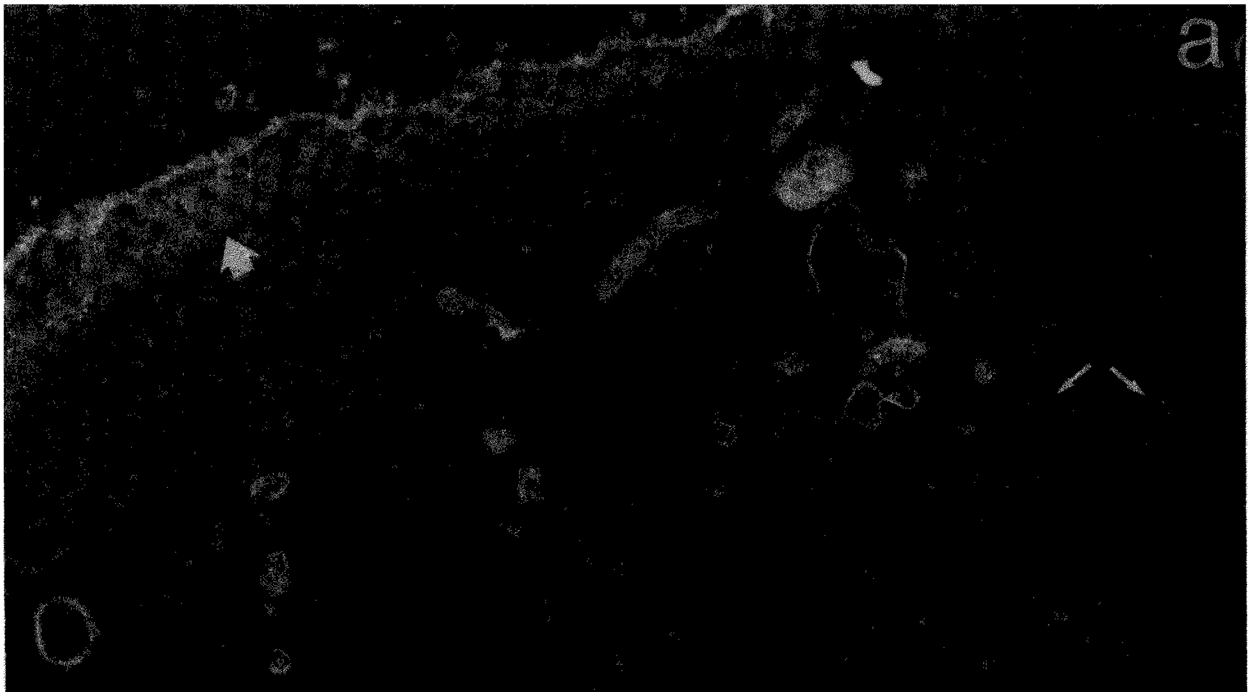

FIGURE 6. Binding by anti-HA-1 to fibers in the tunic from rejection areas. Epon-embedded sections were incubated with anti-HA-1 followed by Protein A-gold (a) to (c), or with normal rabbit serum. (a) Anti-HA-1 incubated section from rejected oozooids, containing a portion of the cuticle (large arrows) and a deeper area containing debris from degenerated test cells in the contact zone (small arrows). Magnification: ×2500. (b) Enlargement of the area highlighted with arrows in (a), showing localization of gold particles to fibers. Magnification: ×5000. (c) Concentration of HA-1 epitopes in an area of the tunic cuticle underlying a cnidarian embedded in the tunic surface (arrows). Magnification: ×4500.

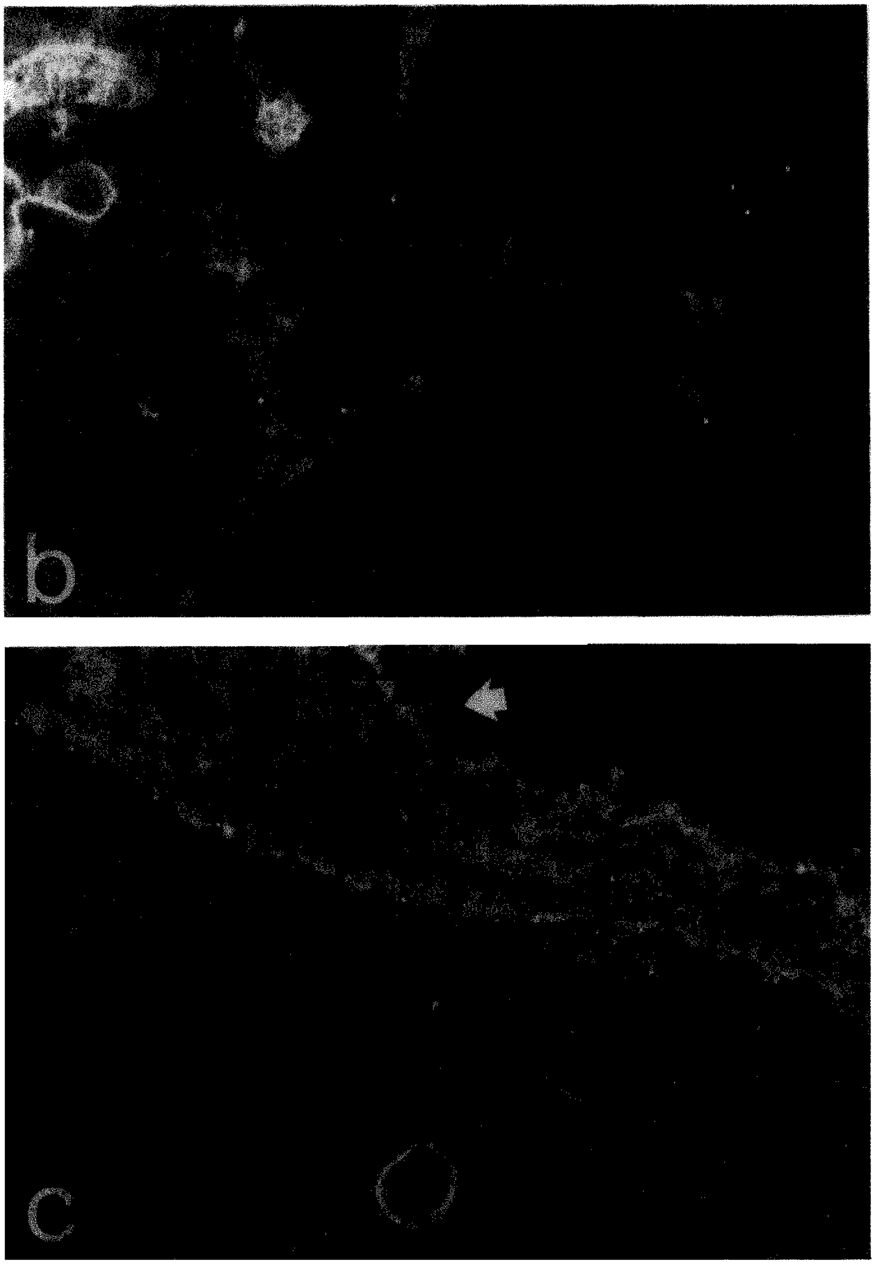

FIGURE 6. Binding by anti-HA-1 to fibers in the tunic from rejection areas. Epon-embedded sections were incubated with anti-HA-1 followed by Protein A-gold (a) to (c), or with normal rabbit serum. (a) Anti-HA-1 incubated section from rejected oozooids, containing a portion of the cuticle (large arrows) and a deeper area containing debris from degenerated test cells in the contact zone (small arrows). Magnification: ×2500. (b) Enlargement of the area highlighted with arrows in (a), showing localization of gold particles to fibers. Magnification: ×5000. (c) Concentration of HA-1 epitopes in an area of the tunic cuticle underlying a cnidarian embedded in the tunic surface (arrows). Magnification: ×4500.

antibody binding to amyloid deposits in all three of the locations identified in Fig. 9 (Liu et al., 1982); Figs. 10(a) to 10(c). When the anti-SAP is replaced by anti-HA-1, an identical binding pattern is seen; Figs. 10(d) and 10(e). Neither anti-SAP nor anti-HA-1 bound to similar tissue sections from two age-matched normal control brains (not shown). Similarly, sections incubated with the second and third reagents (alone or together) displayed no binding (not shown).

### DISCUSSION

### Amyloidlike Fibers Form Barriers Between Allogeneic Colonies

Double-tracked fibers are concentrated in the contacted tunic tissues of rejecting oozooids, where they appear to surround degenerated test cells (Tanaka and Watanabe, 1973a, 1973b); Figs. 5 to 7. The presence of HA-1 epitopes on the

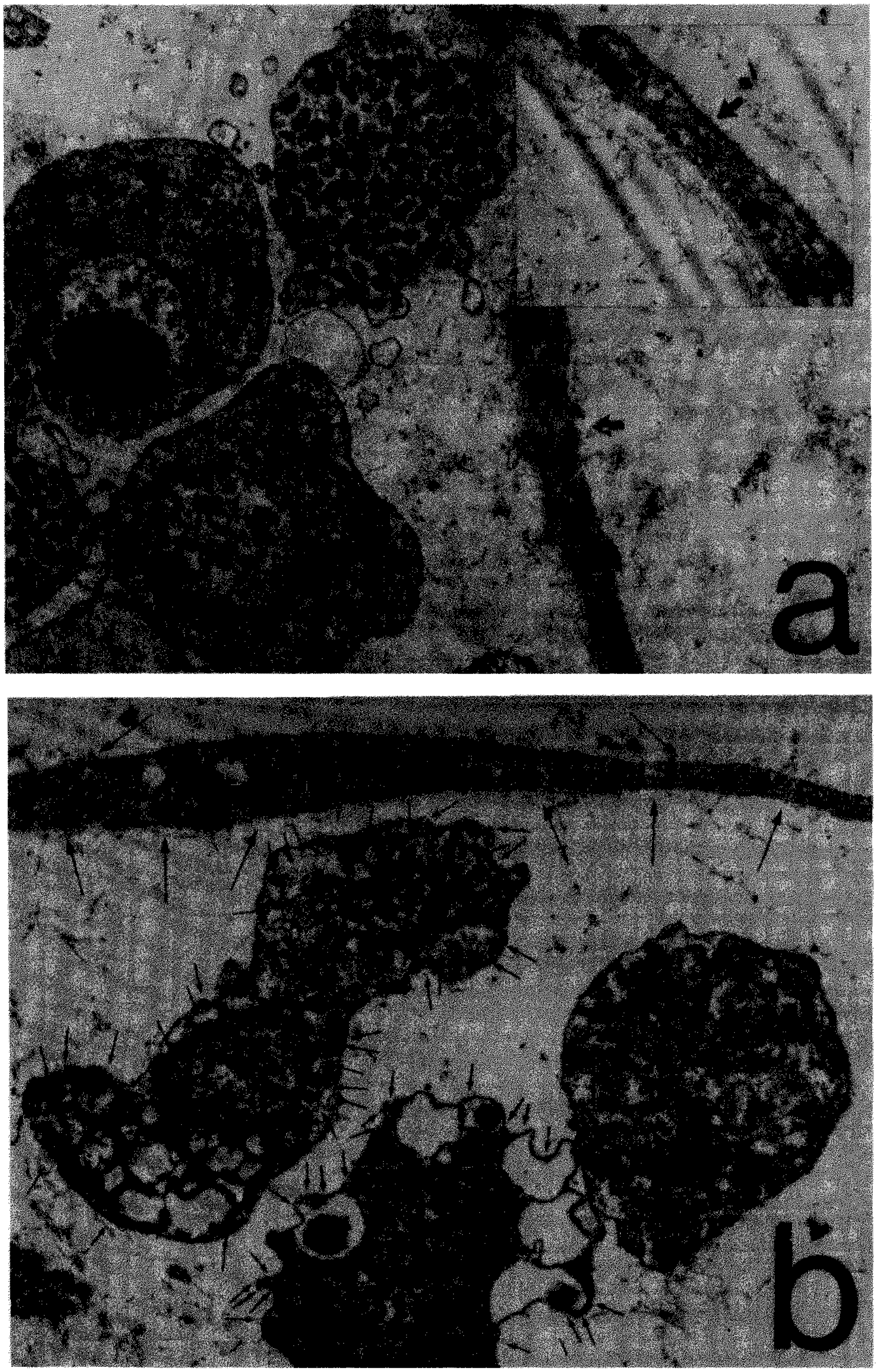

FIGURE 7. Binding by anti-HA-1 to Botrylloides blood cells, vascular endothelial cells, and endothelial cell basement membrane. (a) and (b) Thin sections through ampullae between rejected Botrylloides oozooids, incubated with normal rabbit serum (a), anti SAP (a inset) or anti-HA-1 (b). No gold particles mark the blood cells or ampullar wall after incubation of sections in normal rabbit serum (a), but two of the three blood cells and the ampullar epithelium are heavily labeled after incubation in anti-HA-1 (b). Magnification: ×5000. (a inset) Ampullar epithelium (arrow) from a section incubated with anti-SAP. As with the anti-SAP-incubated methacrylate section in Fig. 5, some antibody binding is evident, but no distinct pattern is seen. Magnification: ×5000.

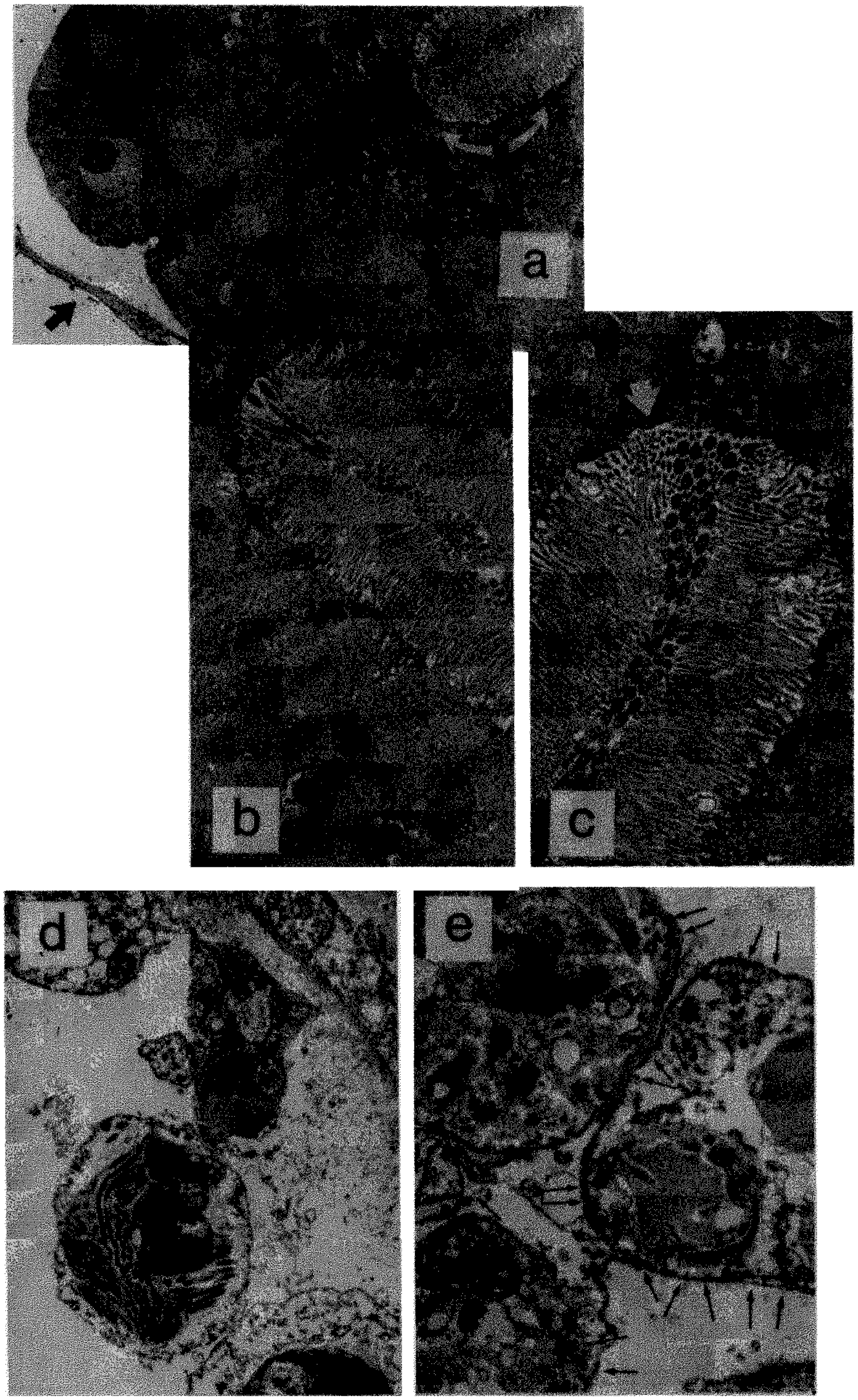

FIGURE 8. HA-1 epitopes in mucus-producing tissues of the branchial basket. (a) Transmission electron micrograph showing the endostylar area of the branchial basket. The white arrows at the upper right mark the elongated endostylar cilia, and the black arrow denotes the overlying tunic cuticle. Magnification: ×1000. (b) and (c) Replicate sections through endostylar tissues and cilia (black arrows), incubated either with normal rabbit serum (b; ×2500) or anti-HA-1 (c; ×3000) followed by Protein A-gold. Heavy concentrations of gold particles are on the surfaces of the languet cilia (white arrows) and in secretory vacuoles of endostylar mucus-producing cells. (e) Sections through the gill-bars of the branchial basket, incubated with anti-SAP. Scattered gold particles are seen in this section, but no pattern of binding is seen. Magnification: ×3000. (f) Similar section incubated with anti-HA-1. HA-1 determinants are present in the cytoplasm and on the surfaces of all three of the cells in this section. Magnification: × 3500.

fibers in these locations and in the tunic cuticle underlying a fouling cnidarian, Figs. 7(a) to 7(c), suggests that HA-1 is a physical component of these structures, and that their deposition is a true amyloidotic process. It is interesting that the blood-cell and epithelial HA-1 epitopes visualized by immunelectron microscopy in Figs. 7 and 8 were accessible to antibody without prior protease treatment of the sections, whereas those in the rejection area in Fig. 5 and in the Alzheimer's disease brain tissue in Fig. 10 were not. Likewise, although double-tracked fibers are

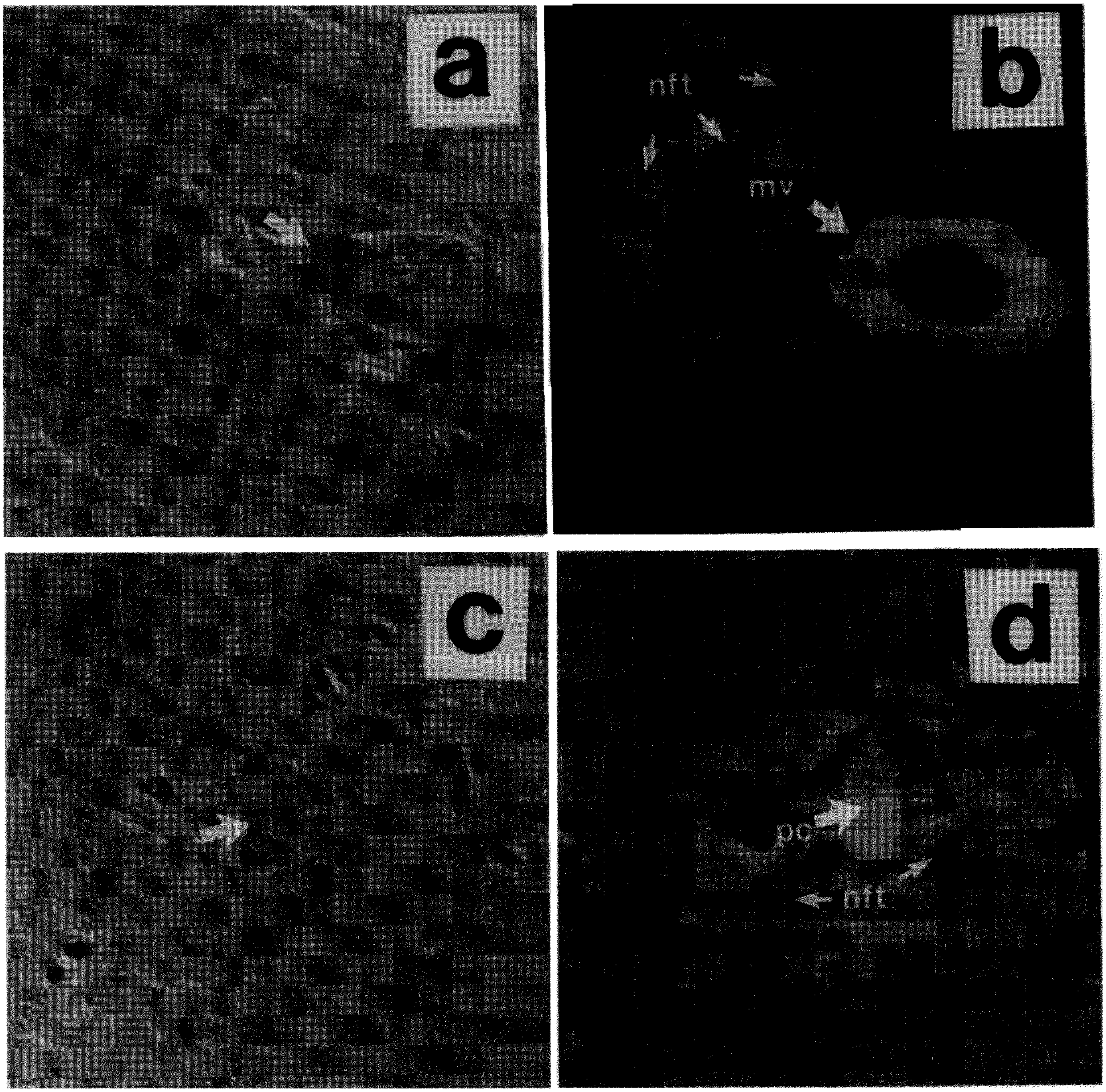

FIGURE 9. Amyloid deposits in Alzheimer's disease brains. (a–d) Congo Red-stained sections from the neocortex of a 72-year-old woman who had died with Alzheimer's disease, showing amyloidotic microvessels, neuritic plaque cores, and degenerating neurites viewed under cross-polárized transmitted light (a and c) and under epifluorescent illumination (b and d). Amyloid in microvessel walls (a) and in the cores of neuritic plaques (c) exhibits apple-green birefringence under cross-polarized light. Illumination of the same fields with ultraviolet light (b and d) reveals amyloid in the surrounding neurites. ("nft"). Magnification: ×400. (See Colour Plate IV at the back of this publication).

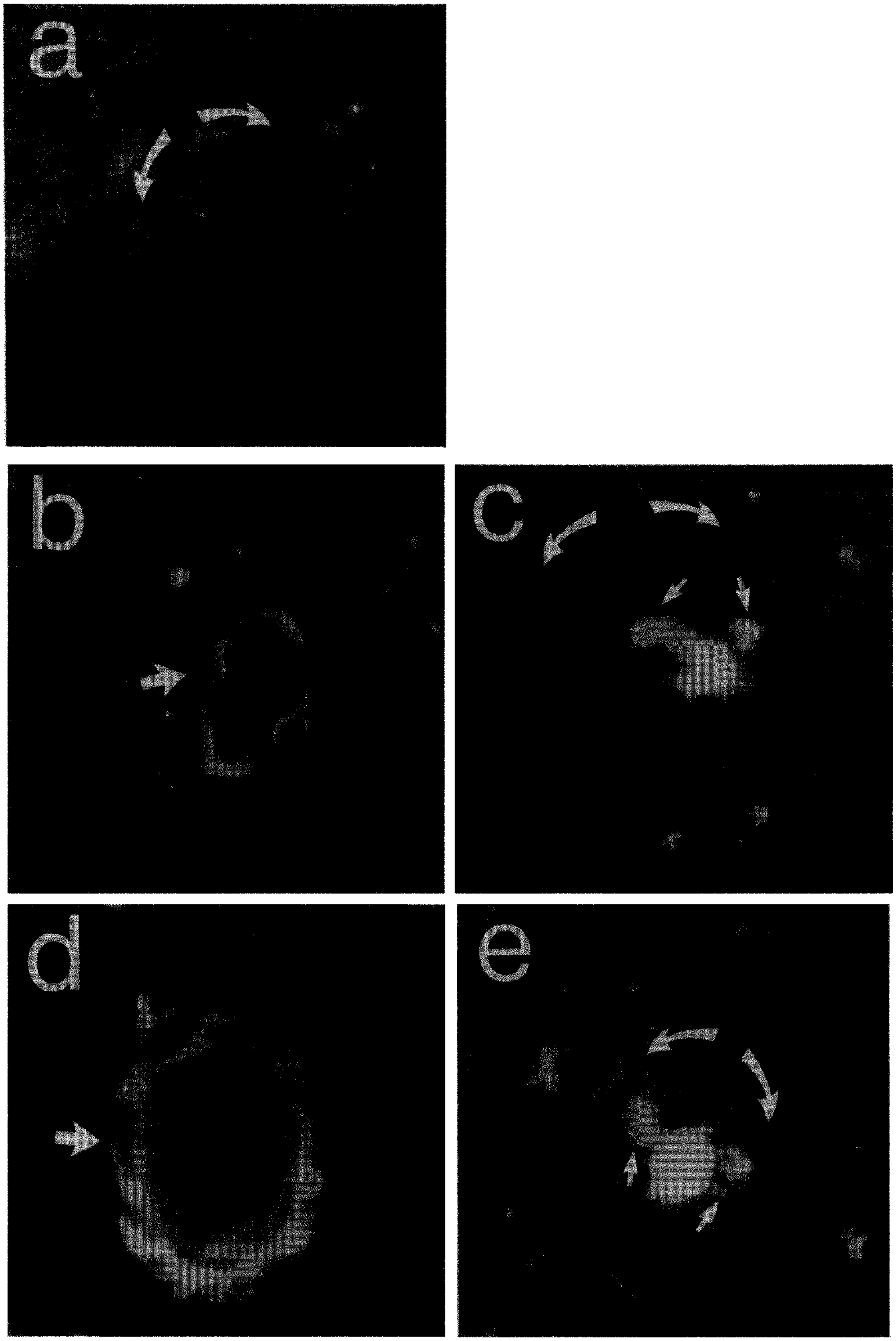

FIGURE 10. Anti-HA-1 binding to Alzheimer's disease amyloid. (a–e) Sections through amyloidotic microvessels and plaques like those in Fig. 9, incubated with normal rabbit serum (a), anti-SAP (b and c), and anti-HA-1 (d and e). As shown previously by others, anti-SAP labels microvessel, plaque, and neurite amyloid (Coria et al., 1988) (b and c). Binding by anti-HA-1 to similar sections reveals an identical pattern (d and e). Arrows in (c) and (e) point to autofluorescent lipofuschin granules surrounding the plaque cores. Magnification of all sections: Magnification of ×400. (See Colour Plate V at the back of this publication).

visible under the entire tunic cuticle in Fig. 6(a), binding by anti-HA-1 occurred only in areas where a defense response had occurred (arrows in Figs. 6(a) to 6(c)). It is possible that the inflammatory reactions accompanying fiber deposition at these sites included local production of proteases capable of unmasking HA-1 epitopes; alternatively, fibers deposited during such reactions may have compositions or secondary structures distinct from those present in normal tissue (Figs. 7 and 8).

The locations of birefringent fiber masses and areas of HA-1 epitope concentration in Figs. 2 to 6 imply that amyloid in tunicates operates for encapsulation of inflammatory foci and for barrier formation against allogeneic challenge. The association of human amyloid deposits with skin lesions in leprosy, with lung granulomas in tuberculosis and in joints in rheumatoid arthritis (Jayalakshmi et al., 1987; Breedveld et al., 1989; Mazur, 1989) suggests that mammalian amyloid serves a similar containment function.

### HA-1 Epitopes Are Present in Protochordate and Mammalian Amyloid

Table 1 and Fig. 1 show that the amino acid composition and three-dimensional structure of the *Botrylloides* HA-1 molecule resemble those of other vertebrate pentraxins (Coe, 1983; Schluter and Ey, 1989). That HA-1 may have functions similar to those of mammalian SAP is suggested by Figs. 5 and 6, which show that HA-1 epitopes are concentrated in areas between rejected oozooids and on fibers deposited near degenerating test cells in these locations, and by Figs. 7 and 8, which show that HA-1 epitopes have a tissue distribution similar to that of CRP and SAP in mammals. Figures 9 and 10 show that anti-HA-1, like anti-SAP (Coria et al., 1988), binds to amyloid fibers within the microvessels of Alzheimer's disease brains, and identifies both the core and neurite amyloid deposits within the senile plaques.

In mammals, both CRP and SAP can serve as *acute-phase* serum proteins (Coe, 1983) whose concentrations increase upon injury or immunological challenge. CRP opsonizes bacteria (Coe, 1983) and fixes complement (Volanakis and Kaplan, 1974; Kilpatrick and Volanakis, 1985). Both SAP and CRP participate in regulation of blood clotting and platelet function (Meyer et al., 1987), and the proteolytic fragments of CRP are potent immunomodulators (Robey et al., 1987). Human and mouse NK and B cells have monomeric surface CRP and SAP that comodulates with Fc receptors for immunoglobulins (James et al., 1983; Bray et al., 1988). NK cell surface CRP appears to participate in target cell killing, by mechanisms that remain to be defined (Baum et al., 1983). Finally, mammalian SAP, like HA-1 (Coombe et al., 1984a), is a serum lectin that mediates calcium-dependent hemagglutination via carbohydrates on red cell surfaces (Hamazaki, 1988). The host defense functions of pentraxins intersect with those of adaptive immunity on several levels, and comparisons of pentraxins and immunoglobulins suggest a limited structural similarity between their molecules as well (Vasta et al., 1986; Schluter and Marchalonis, 1989). Isolation and sequencing of the HA-1 genes of tunicates will allow positive identification of the HA-1 lectin as a pentraxin. Identification of cell surface molecules associated with it then may offer clues to relationships between the pentraxin/immunoglobulin superfamilies in the early evolution of adaptive immunity.

### MATERIALS AND METHODS

### Animals

Colonies of *Botryllus schlosseri* and *Botrylloides violaceous* were collected from local harbors and maintained in the laboratory in aquaria under aeration. Embryos and mature tadpole larvae were removed by dissection and allowed to metamorphose, after which the oozooids were removed carefully and reattached in pairs to glass or plastic surfaces, as described previously (Scofield et al., 1982). After the transplantation reactions (fusion or rejection) were completed, the rejected pairs were selected, removed with razor blades, and embedded in methacrylate for histochemistry and immunofluorescence studies, or in Epon or Lowicryl resin for transmission and immunoelectron microscopy. For both light and electron microscopy, sections were cut from embedded blocks with an ultramicrotome.

### Histochemical Staining

For visualization of birefringence in rejection interfaces between oozooids, sections were stained with alcoholic basic fuchsin (Puchtler et al., 1962) (tunicate tissue) or Congo Red (Skinner et al., 1982) (tunicate and human tissue), and photographed under cross-polarized light (Puchtler et al., 1962; Skinner et al., 1982).

### Proteins and Antibodies

HA-1 was prepared from *Botrylloides* hemolymph, as described previously (Coombe et al., 1984a; Schluter and Ey, 1989), and affinity-purified anti-HA-1 prepared from serum of a rabbit immunized with HA-1 in complete Freund's adjuvant (Coombe et al., 1984b). The blood cell-binding characteristics of this reagent have been described (Coombe, 1983). Affinity-purified rabbit antihuman SAP was from Accurate Chemical Corporation. For visualization of antibody binding to sections, biotinylated goat antirabbit immunoglobulin and avidin-D fluorescein isothiocyanate were obtained from Vector Laboratories, Fluoresbrite beads purchased from Polysciences, and Staph Protein A bought from Sigma. The Protein A-Gold used was Auroprobe G10 from Janssen Life Sciences.

### Histochemistry and Immunohistochemistry with Tunicate Tissues

Tunicate oozooid pairs were fixed briefly in 1% formalin and embedded in methacrylate (Immuno-Bed; Polysciences). Sections were cut in a plane that incorporated both oozooid bodies and the rejection area between them. Congo Red and basic fuchsin staining were done with unfixed sections after a brief hydration step in distilled water. For assays of antibody binding to tunicate tissues embedded in methacrylate, all sections were incubated for 1 hr in anti-HA-1, anti-SAP, or normal rabbit serum at a 1:20 dilution. Areas of antibody binding were visualized by their fluorescence under ultraviolet illumination after sequential incubation in biotinylated goat antirabbit Ig (1:50) and mixed Protein A- and avidin D-conjugated Fluoresbrite beads (Polysciences). All sections in Fig. 5 had been preincubated for 30 min in a trypsin solution prior to addition of normal rabbit serum or specific antibodies.

### Human Tissues

The test tissue for light microscopic detection of antibody binding to human amyloid was human brain removed at autopsy and fixed in 10% neutral buffered formalin. Blocks were embedded in paraffin by methods used in routine pathological work. Confirmation of Alzheimer's disease was by standard pathological criteria, including identification of Congophilic microvascular and neuritic plaques (Vinters and Gilbert, 1983). As with the tunicate tissues, all sections were first incubated briefly in a dilute protease solution (in this case, with pepsin; Coria et al., 1988), followed by 1:50 dilutions of the test antibodies or normal rabbit serum. This was followed sequentially by biotinylated goat antirabbit Ig and avidin-D fluorescein.

### Electron Microscopy

For transmission electron microscopy, rejected oozooids were fixed in 2% glutaraldehyde, embedded in Epon, stained with osmium tetroxide and uranyl acetate, and sectioned in areas previously selected by light microscopy for birefringence. HA-1 molecules were visualized on a Formvar-coated grid after negative staining with phosphotungstic acid, as described previously for vertebrate CRP and SAP preparations (Pepys et al., 1978). Observation and photography were done with a JOEL-100X electron microscope.

### Amino Acid Composition

Amino acid analysis of HA-1 was by reverse-phase HPLC using the Pico-Tag method (Bidlingmeyer et al, 1984).

### ACKNOWLEDGMENTS

This work was supported by grants to V.S. from the American Cancer Society (#IM-403 and IM-403A), the American Heart Association (Los Angeles Branch), and the California Institute for Cancer Research (UCLA). Alzheimer's disease brain tissue was provided by Dr. Harry Vinters and Ms. Diana Lenard Secor of the Department of Pathology, UCLA School of Medicine. We thank Drs. Kenneth Kustin, Jonathan Coe, Gerardo Vasta, and Henry Gewurz for essential discussions, and we acknowledge with gratitude contributions by Drs.

Martin Poenie, David Epel, and Irving Weissman to early phases of this study.

*(Received July 25, 1991)*

*(Accepted February 12, 1992)*

### REFERENCES

Baum L.L., James K.K., Graziano R., and Gewurz H. (1983). Possible role for C-reactive protein in the human natural killer cell response. J. Exp. Med. **157:** 301–311.

Bidlingmeyer B.A., Cohen S.A., and Tarvin T.L. (1984) Rapid analysis of amino acids using pre-column derivatization. J. Chromatog. **336:** 93–104.

Braun J., Schultek T., Tegtmeier K.F., Florenz A., Rohnde C., and Wood W.G. (1986). Luminometric assays of seven acute-phase proteins in minimal volumes of serum, plasma, sputum, and bronchioalveolar lavage. Clin. Chem. **32:** 743–747.

Bray R.A., Samberg N.L., Gewurz H., Potempa L.A., and Landay A.L. (1988). C-reactive protein antigenicity on the surface of human peripheral blood lymphocytes; characterization of lymphocytes reactive with anti-neo-CRP. J. Immunol. **140:** 4271–4276.

Breedveld F.C., Markusse H.M., and McFarlane J.D. (1989). Subcutaneous fat biopsy in the diagnosis of amyloidosis secondary to chronic arthritis. Clin. Exp. Rheumatol. **7:** 407–410.

Coe J.E. (1983). Homologs of C-reactive protein: A diverse family of proteins with similar structure. Contemp. Top. Molec. Immunol. **9:** 211–239.

Coombe D.R. (1983) Recognition of foreign particles by the protochordate *Botrylloides leachii*. Ph.D. thesis, Department of Zoology, University of Adelaide.

Coombe D.R., Ey P.L., and Jenkin C.R. (1984a). Ascidian hemagglutinins: Incidence in various species, binding specificity and preliminary characteristics of selected agglutinins. Comp. Biochem. Physiol. **77B:** 811–819.

Coombe D.R., Ey P.L., and Jenkin C.R. (1984b). Particle recognition by hemocytes from the colonial ascidian *Botrylloides leachi*: Evidence that the HA-2 lectin is opsonic. J. Comp. Physiol. **B154:** 509–521.

Coria F., Castano E., Prelli F., Larrondo-Lillo M., van Duinen S., Shelanski M.L., and Frangione B. (1988). Isolation and characterization of amyloid P component from Alzheimer's disease and other types of cerebral amyloidosis. Lab. Invest. **58:** 454–458.

Deck J.D., Hay E.D. and Revel J.P. (1966). Fine structure and origin of the tunic of *Perophera viridis*. J. Morphol. **120:** 267–280.

Dyck R.F., Lockwood C.M., Kershaw M., McHugh N., Duance V., Baltz M., and Pepys M.B. (1980). Amyloid P component is a component of normal human glomerular basement membrane. J. Exp. Med. **152:** 1162–1164.

Ellis L.C., and Youson J.H. (1989). Ultrastructure of the pronephrotic kidney in the upstream migrant sea lamprey *Petromyzon marinus* L. Amer. J. Anat. **185:** 449–453.

Fernandez-Moran H., Marchalonis J.J., and Edelman G.M. (1968). Electron microscopy of a hemagglutinin from *Limulus polyphemus*. J. Mol. Biol. **32:** 467–469.

Goodbody I. (1974). The physiology of ascidians. Adv. Mar. Biol. **12:** 1–149.

Hamazaki H. (1988). Calcium-mediated hemagglutination by serum amyloid P component and the inhibition by specific glycosaminoglycans. Bioch. Biophys. Res. Comm. **150:** 212–218.

Holck M., Husby G., Sletten K., and Natvig J.B. (1979). Isolation of AP from amyloid fibrils. Scand. J. Immunol. **10:** 55–65.

Inoue S., LeBlond C.P., Grant D.S., and Rico P. (1986). The microfibrils of connective tissue. II. Immunohistochemical definition of the amyloid P component. Amer. J. Anat. **176:** 139–152.

Inoue S. and LeBlond C.P. (1986). The microfibrils of connective tissue. I. Ultrastructure. Amer. J. Anat. **176:** 121–138.

James K., Baum L., Adamowski C., and Gewurz H. (1983). C-reactive protein antigenicity on the surface of human lymphocytes. J. Immunol. **131:** 2930–2934.

Jayalakshmi P., Looi L.M., Lim K.J., and Rajogopalan K. (1987). Autopsy findings in 35 cases of leprosy in Malaysia. Int. J. Leprosy and Mycobact. Dis. **55:** 510–514.

Katow H., and Watanabe H. (1980). Processes of fusion in *Botryllus primigenus* Oka. Devel. Biol. **76:** 1–35.

Kilpatrick J.M., and Volanakis J.E. (1985). Opsonic properties of C-reactive protein. Stimulation by phorbol myristate acetate enables human neutrophils to phagocytose C-reactive protein coated cells. J. Immunol. **134:** 3364–3370.

Liu T.Y., Robey F.A., and Wang C.M. (1982). Structural studies on C-reactive protein. Ann. N.Y. Acad. Sci. **389:** 151–164.

Maudsley S., and Pepys M.B. (1987). Immunochemical crossreactions between pentraxins of different species. Immunology **62:** 17–22.

Mazur P.E. (1989). Amyloidosis lesions of the digestive organs in patients with tuberculosis and chronic nonspecific lung diseases. Prob. Tuberk. **11:** 46–49.

Meyer K., Smith R., and Williams E.D. (1987). Inhibition of fibrin polymerization by serum amyloid P component and heparin. Thromb. Haem. **57:** 345–348.

Miyakawa T. (1988). Ultrastructural study of senile plaques and microvessels in the brain in Alzheimer's disease. *In*: Immunology and Alzheimer's disease, Pouplard-Barthelaiz A., and Christen J.T., Eds. (New York: Springer-Verlag), pp. 42–54.

Nguyen N.Y., Suzuki A., Boykins R.A., and Liu T.Y. (1986a). The amino acid sequence of *Limulus* C-reactive protein. Evidence of polymorphism. J. Biol. Chem. **261:** 10456–10465.

Nguyen N.Y., Suzuki A., Cheng S.M., Zon G., and Liu T.Y. (1986b). Isolation and characterization of *Limulus* C-reactive protein genes. J. Biol. Chem. **261:** 10450–10455.

Oka H., and Watanabe H. (1957). Colony specificity in colonial ascidians as tested by fusion experiments. Proc. Jpn. Acad. Sci. **33:** 657–672.

Pepys M.B. (1988). Amyloidosis. *In*: Immunologic Diseases, Samter M., Austin K.F., Claman, H.N., Frank M.M., and Talmage D.W., Eds. (Boston: Little, Brown), pp. 000–000.

Pepys M.B., Dash A.C., Fletcher T.C., Richardson, N., Munn E.A., and Feinstein A. (1978). Analogs in other mammals and in fish of human palsma proteins, C-reactive protein and amyloid P component. Nature **273:** 168–170.

Pepys M.B., Fletcher T.C., Milstein C.P., Feinstein A., March J.F., Buttress N., Munn E.A. and deBeer F.C. (1982). Isolation from plaice (*Pleuronectes platessa* L.) serum of C-reactive protein (CRP) and amyloid P component (SAP) and their characterization as homologues of human CRP and SAP. Biochem. Biophys. Acta. **704:** 123–133.

Puchtler H., Sweat F., and Levine M. (1962). The binding of Congo red by amyloid. J. Histochem. Cytochem. **10:** 355–368.

Robey F.A., Ohura K., Futaki S., Fujii N., Yajima H., Goldman N., Jones K.D., and Wahl S. (1987). Proteolysis of human C-reactive protein produces peptides with potent immunomodulating activity. J. Biol. Chem. **262:** 7053–7057.

Robey R.A., and Liu T.Y. (1981). Limulin: A C-reactive protein from *Limulus polyphemus*. J. Biol. Chem. **256:** 969–975.

Schluter S.F., and Ey P.L. (1989). Purification of three lectins from the hemolymph of the ascidian *Botrylloides leachi*. Comp. Biochem. Physiol. **93B:** 145–152.

Schluter S., and Marchalonis J.J. (1989). Immunoproteins in evolution. Dev. Comp. Immunol. **13:** 285–301.

Scofield V.L., Schlumpberger J.M., West L.A., and Weissman I.L. (1982). Protochordate allorecognition is controlled by a MHC-like gene complex. Nature **295:** 499–502.

Scofield V.L., and Nagashima L.S. (1982). Morphology and genetics of rejection reactions between oozooids of the tunicate *Botryllus schlosseri*. Biol. Bull. **165:** 733–745.

Siripont J., Tebo J.M., and Mortensen R.F. (1988). Receptor-mediated binding of the acute-phase reactant mouse serum amyloid P component (SAP) to macrophages. Cell. Immunol. **117:** 239–252.

Skinner M., Pepys M.B., Cohen A.S., Heller L.M., and Lian J.B. (1980). Studies of amyloid protein AP. *In*: Amyloid and amyloidosis, Glenner G.G., Pinho de Costa P., and de Frietas F., Eds. (Amsterdam: Excerpta Medica), pp. 384–391.

Skinner M., Sipe J.D., Yood R.A., Shirahama T., and Cohen A.S. (1982). Characterization of P-component (AP) isolated from amyloidotic tissue: Half-life studies of human and murine AP. Ann. N.Y. Acad. Sci. **389:** 190–198.

Tanaka K., and Watanabe H. (1973a). Allogeneic inhibition in a compound ascidian (*Botryllus primigenus* Oka). Processes and features of nonfusion reaction. Cell. Immunol. **7:** 410–426.

Tanaka K., and Watanabe H. (1983b). Allogeneic inhibition in a compound ascidian (*Botryllus primigenus* Oka). Cellular and humoral responses in nonfusion reaction. Cell. Immunol. **7:** 427–443.

Vasta G.R., Marchalonis J.J., and Kohler H. (1986). Invertebrate recognition protein cross-reacts with an immunoglobulin idiotype. J. Exp. Med. **156:** 1270–1276.

Vinters H.V., and Gilbert J.J. (1983). Cerebral amyloid angiopathy: Incidence and complications in the aging brain. II. The distribution of amyloid vascular changes. Stroke **14:** 924–928.

Volanakis J.E., and Kaplan M.H. (1974). Interaction of C-reactive protein complexes with the complement system. II. Consumption of guinea pig complement by CRP complexes: Requirement for human C1q. J. Immunol. **113:** 9–18.

Weissman I.L., Saito Y., and Rinkevich B. (1990). Allorecognition histocompatibility in a protochordate species: Is the relationship to MHC somatic or structural? Immunol. Rev. **113:** 227–241.

